# Supplementary material for: A Citizen Science Approach to Determine Physical Activity Patterns and Demographics of Greenway Users in Winston-Salem, North Carolina
Source: Int J Environ Res Public Health. 2019 Aug 29;16(17):3150. doi: 10.3390/ijerph16173150 (PMC6747415; doi:10.3390/ijerph16173150)
Supplement: Supplementary file 1 [file ijerph-16-03150-s001.pdf]

Physical Activity Observation Form

Location: □ Brushy Fork □ 4th Street □ Salem Lake □ West End □ Strollway

**Observer name:**	**Date:** ___/___/______	**Start time:** ___:___	**End time:** ___:___

**Number of Walkers/Runners**	**YOUTH (2-20 years)**		**ADULT (21-59 years)**		**SENIOR (60+ years)**	
	**Moderate**	**Vigorous**	**Moderate**	**Vigorous**	**Moderate**	**Vigorous**
**Female**						
**White**						
**Black**						
**Asian**						
**Hispanic**						
**Other/Unsure**						
**Male**						
**White**						
**Black**						
**Asian**						
**Hispanic**						
**Other/Unsure**						

**Number of Bike Riders**	**YOUTH (2-20 years)**		**ADULT (21-59 years)**		**SENIOR (60+ years)**	
	**Moderate**	**Vigorous**	**Moderate**	**Vigorous**	**Moderate**	**Vigorous**
**Female**						
**White**						
**Black**						
**Asian**						
**Hispanic**						
**Other/Unsure**						
**Male**						
**White**						
**Black**						
**Asian**						
**Hispanic**						
**Other/Unsure**						

Activity Level: Moderate = moving at a slow, casual pace; Vigorous = engaged in an activity more vigorous than an ordinary walk

Go to https://ctsi.wakehealth.edu/community/what-we-do/citizen-science to input data or scan QR Code

Conditions of Target Area			
Is the area accessible (e.g., not locked or rented to others)?	______Yes	______No	
Is the area usable (e.g., not excessively wet or windy)?	______Yes	______No	
Is equipment available in the area (e.g., sport balls available)?	______Yes	______No	
Is the area supervised (e.g., park-related personnel present)?	______Yes	______No	
Is there organized activity in the area (e.g., group bike ride)?	______Yes	______No	
Is the area sufficiently lit (if dawn/dusk/nighttime)?	______Yes	______No	______NA
Amount of sun or shade in the target area:	______Mostly shade	_____Shade/ sun mix	______Mostly sunny

General comments/observations:

Go to https://ctsi.wakehealth.edu/community/what-we-do/citizen-science to input data or scan QR Code
